# Adenoviral Vectors for Hemophilia Gene Therapy

**Published:** 2013-04-30

**Authors:** N Brunetti-Pierri, Philip Ng

**Affiliations:** 1 Telethon Institute of Genetics and Medicine, Naples, Italy; 2 Department of Translational Medicine, Federico II University of Naples, Italy; 3 Department of Molecular and Human Genetics, Baylor College of Medicine, Houston, TX, USA

**Keywords:** Hemophilia A, Hemophilia B, Factor VIII, Factor IX, Gene therapy, Adenovirus, Viral vectors, Helper-dependent adenoviral vectors

## Abstract

Hemophilia is an inherited blood clotting disorder resulting from deficiency of blood coagulation factors. Current standard of care for hemophilia patients is frequent intravenous infusions of the missing coagulation factor. Gene therapy for hemophilia involves the introduction of a normal copy of the deficient coagulation factor gene thereby potentially offering a definitive cure for the bleeding disorder. A variety of approaches have been pursued for hemophilia gene therapy and this review article focuses on those that use adenoviral vectors.

## Introduction

Hemophilia is a group of genetic disorders that impairs blood clot formation. Hemophilia A is a deficiency in coagulation factor VIII (FVIII) and hemophilia B is a deficiency in coagulation factor IX (FIX). Current standard of care for hemophilia patients is protein replacement therapy whereby patients are intravenously infused with the missing coagulation factor and this is effective in limiting acute bleeds and reducing morbidity and mortality, as well as preventing long term disability, pain, and reduced range of motion in joints for both hemophilia A and B patients [1,2]. However, the short half-life of FVIII and FIX require multiple weekly intravenous infusions to achieve hemostasis [3,4]. This high frequency is inconvenient and costly and thus protein replacement therapy is often given in response to bleeds rather than prophylactically. In addition, protein replacement therapy presents several problems including complications in venous access, inhibitor formation, allergic reactions, and thrombosis [5].

Given these disadvantages, the motivation to develop gene therapy for hemophilia is high and significant efforts have been made towards this goal in the last two decades. Hemophilia A and B are attractive candidates for gene therapy because: 1) even low levels (1% of normal activity) of clotting factors are therapeutic, as established by clinical studies with infusions of recombinant factor [2]; 2) FIX can be produced by a variety of tissues and still retain its biological activity [6]; 3) small and large disease animal models are available to investigate experimental treatments; 4) determination of clinically relevant endpoints is straightforward and unequivocal using well-characterized clotting assays. Despite the clinical similarities, there are a number of important differences between hemophilia A and B. First, the prevalence of hemophilia A is ~10 fold higher than hemophilia B [7] and this could facilitate patient enrolment for clinical trials. Second, the FIX cDNA is relatively small (~1.5 kb) and thus can be accommodated into most viral vectors, while the larger 7 kb FVIII cDNA (or even at 4.4 kb for the B-domain deleted construct) can limit vector choice. Third, for protein-based therapy, development of neutralizing antibodies (called inhibitors) occurs much more frequently against FVIII (7–52%) than FIX (1–3%) [8], thus favoring hemophilia B for experimental treatments such as gene therapy. On the other hand, normal circulating levels of FVIII (100 to 200 ng/ml) are much lower than normal circulating levels of FIX (3,000 to 5,000 ng/ml), thus levels of protein expression required for clinical benefit may be less for FVIII thus perhaps necessitating lower vector doses which would improve safety.

A variety of gene and cell therapy strategies have been investigated for hemophilia gene therapy and many of these are covered by other review articles in this journal’s series. This review will focus solely on adenoviral vectors for hemophilia gene therapy.

## The Adenovirus

**Figure 1:**
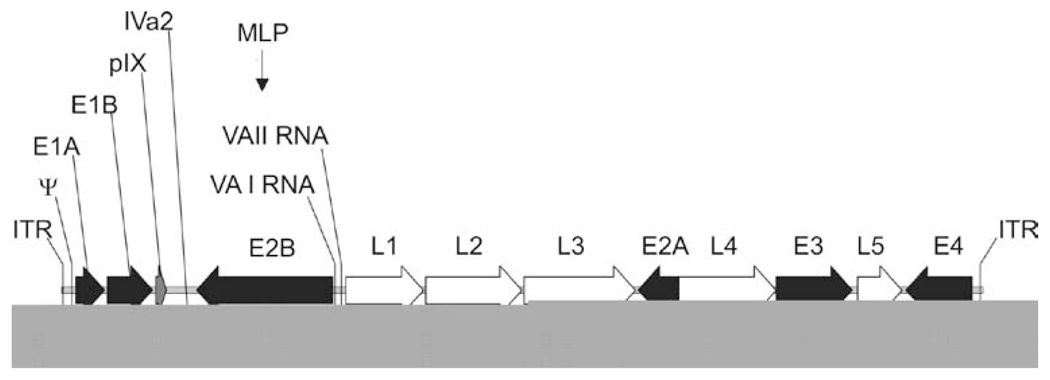
Map of human adenovirus serotype 5. The ~36 kb genome is divided into four early region transcription units, E1 E4, and five families of late mRNA, L1–L5, which are alternative splice products of a common late transcript expressed from the major late promoter (MLP). Four smaller transcripts, pIX, IVa, and VA RNA’s I and II, are also produced. The 103 bp inverted terminal repeats (ITRs) are located at the termini of the genome and are involved in viral DNA replication, and the packaging signal (ψ) located from nucleotides 190 to 380 at the left end is involved in packaging of the genome into virion capsids.

The adenovirus (Ad) has a non-enveloped icosahedral capsid of ~100 nm containing a linear double-stranded DNA genome of ~ 36 kb [9]. Of the ~50 serotypes of human Ad, the most extensively characterized and thus vectorized are serotypes 2 (Ad2) and 5 (Ad5) of subgroup C. The 36 kb genome of Ad2 and Ad5 is flanked by cis-acting inverted terminal repeats (ITRs) which are required for viral DNA replication. A cis-acting packaging signal (Ψ), required for the encapsidation of the Ad genome is located near the left ITR (Figure 1). The Ad genome can be roughly divided into two sets of genes: the early region genes, E1A, E1B, E2, E3 and E4, are expressed before DNA replication and the late region genes, L1 to L5 are expressed to high levels after initiation of DNA replication. The E1A transcription unit is the first early region to be expressed during viral infection and it encodes two major E1A proteins that are involved in transcriptional regulation of the virus and stimulation of the host cell to enter an S phase-like state. The two major E1B proteins are necessary for blocking host mRNA transport, stimulating viral mRNA transport and blocking E1A-induced apoptosis. The E2 region encodes proteins required for viral DNA replication and can be divided into two subregions; E2a encodes the 72-kD DNA-binding protein and E2b encodes the viral DNA polymerase and terminal protein precursor (pTP). The E3 region, which is dispensable for virus growth in cell culture, encodes at least seven proteins most of which are involved in host immune evasion. The E4 region encodes at least six proteins, some functioning to facilitate DNA replication, enhance late gene expression, and decrease host protein synthesis. The late region genes are expressed from a common major late promoter and are generated by alternative splicing of a single transcript. Most of the late mRNAs encode virion structural proteins. In addition to early and late region genes, four other small transcripts are also produced. The gene encoding protein IX (pIX) is colinear with E1B but uses a different promoter and is expressed at an intermediate time, as is the pIVa2 gene. Other late transcripts include the RNA polymerase III transcribed VA RNA I and II.

## The Adenoviral Vector

The human Ad was one of the first viruses to be developed into a gene transfer vector for gene therapy. This was accomplished by deleting the viral E1 genes to render the vector replication deficient. These first generation Ad vectors (FGAd), produced in an E1-complementing cell line [10], are highly efficient at transducing target cells *in vitro* and *in vivo* and mediate very high level transgene expression. Thus some of the earliest studies in hemophilia gene therapy were conducted with FGAds. All these early studies showed that Ad can mediate very high, supratherapeutic levels of FIX *in vivo*. For example, administration of FGAd expressing FIX achieved complete correction of the bleeding diathesis in FIX-deficient dogs with levels of plasma FIX reaching 300% of normal [11]. However, correction was transient and FIX levels declined after a few days with therapeutic levels persisting for only 1–2 months. Similarly, intravascular administration of FGAd expressing hFIX into rhesus macaque resulted in high level hFIX at 4 days post-injection but declined to undetectable levels by 2–3 weeks [63].

**Figure 2:**
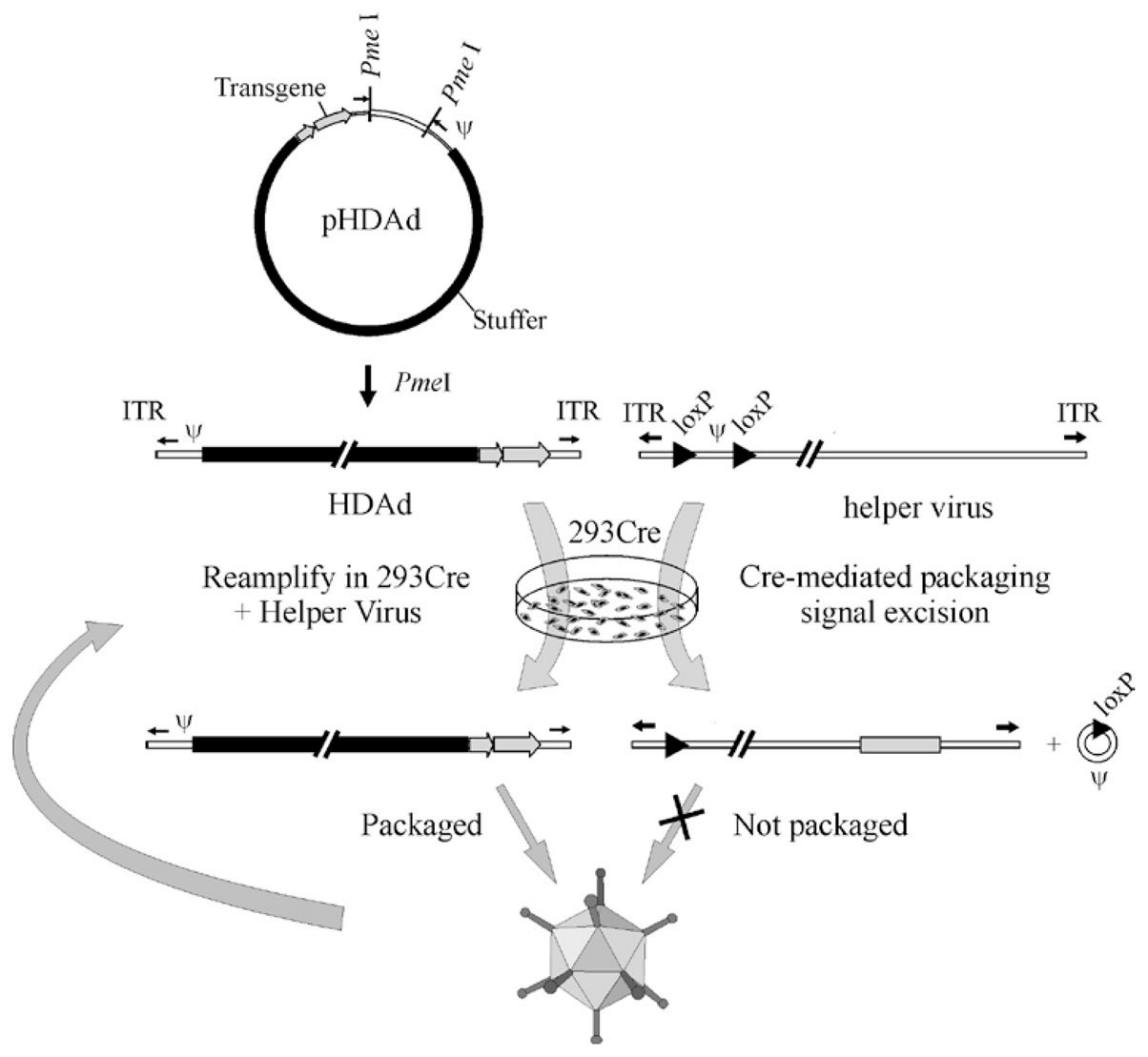
The Cre/loxP system for generating HDAds. The HDAd contains only ~500 bp of *cis*-acting Ad sequences required for DNA replication (ITRs) and packaging (ψ); the remainder of the genome consists of the desired transgene and non-Ad *stuffer* sequences. The HDAd genome is constructed as a bacterial plasmid (pHDAd) and is liberated by restriction enzyme digestion (e.g., *Pme*I). To rescue the HDAd, the liberated genome is transfected into a Cre expressing, E1 complementing cell line and infected with a helper virus, an FGAd bearing a packaging signal (ψ) flanked by loxP sites. Cre-mediated excision of ψ renders the helper virus genome unpackageable, but still able to provide all of the necessary *trans*-acting factors for propagation of the HDAd. The titer of the HDAd is increased by serial coinfections with the HDAd and the helper virus.

The reason that FGAd provided only transient transgene expression was the erroneous assumption that deletion of E1 would silence the rest of the viral genes still present in the vector backbone. Instead, low level viral gene expression in transduced cells flagged them for elimination by the adaptive cellular immune response thus resulting in transient transgene expression and chronic toxicity [12–15]. Processing of proteins derived from the viral capsid shell has also been implicated in this phenomenon [16]. Clearly, further silencing viral gene expression was needed to avoid adaptive immune response against transduced target cells. Two strategies were pursued to achieve this objective. One approach involved deletion of additional viral early genes such as E2 and E4. These second generation Ad were easily propagated in cell lines engineered to express the missing E2 or E4 functions. The other approach was to delete all of the adenoviral protein coding genes from the vector thus eliminating expression of viral proteins from the vector [17]. These vectors require a helper-virus for propagation and are thus called helper-dependent adenoviral vectors (HDAd) (Figure 2). An additional benefit of the large deletion of all viral genes from HDAds is their tremendous cloning capacity; up to 37 kb of foreign sequences can be inserted so that the large full-length hFVIII cDNA can be easily accommodated within HDAd. Production of HDAd is beyond the scope of this review but is detailed elsewhere [18].

Reddy et al. [19] compared the efficacy and safety of hemophilia A gene therapy using a second generation Ad vector (E1,E2a,E3-deleted) and a HDAd, both containing the identical B-domain deleted human FVIII expression cassette. In this study, hemophilia A mice were intravenously injected with 6×10^10^ particles of each vector. Plasma hFVIII levels in mice treated with HDAd peaked at 2 weeks post-injection and were 10-fold higher than levels achieved using the second generation Ad. Expression of hFVIII in HDAd injected mice was sustained for at least 40 weeks although a ~10-fold decrease in plasma levels was observed between weeks 2 and 40. In contrast, plasma hFVIII levels in second generation Ad injected mice rapidly decreased to below the limit of detection by 12 weeks. At a dose of 3×10^11^ particles (1.5×10^13^ particles/kg) both vectors induced hepatotoxicity as evident by ~10-fold increase in AST and ALT levels measured 1 day after vector administration. These levels returned to baseline by day 3 post-injection. However, by day 7, animals injected with second generation Ad showed a 10-fold elevation in AST and ALT levels whereas those injected with HDAd remained at baseline levels. AST and ALT levels did not return to baseline levels until day 28 in the second generation Ad injected animals. These results suggested that the initial increase in liver transaminases observed at day 1 was caused by direct toxicity of virion capsid proteins from both vector types. The toxicity observed at day 7 and beyond for second generation, but not HDAd, may have been due to viral gene expression from the backbone of the second generation Ad. This study clearly demonstrated that HDAd were superior to second generation Ad in the murine hemophilia A model with respect to safety and efficacy. Reddy et al. also reported that neither HDAd nor second generation Ad resulted in the development of anti-hFVIII antibodies [19]. This is in contrast to what was reported by Balague et al. [20] who reported inhibitor development in some, but not all, hemophilia A mice injected with HDAd expressing hFVIII. However, like Reddy’s study, in those mice that did not develop inhibitors, long-term hFVIII expression was observed. It is not known why inhibitors were seen in one study but not another and may be related to the genetic background of the mice. However, it is interesting to note that endogenous Ad-mediated expression of FVIII was much less immunogenic than recombinant protein administration [21]. Regardless, tolerance to human FVIII can be achieved by neonatal injection of HDAd vector in hemophilia A mice [22].

## Preclinical and Clinical Studies

The safety and efficacy for HDAd mediated hemophilia gene therapy has also been evaluated in the large hemophilic canine model [23–26]. In the case of FIX-deficient dogs, systemic intravascular injection of 3×10^12^ vp/kg of HDAd expressing canine FIX resulted in reducing whole blood clotting time from a pre-treatment of >60 min to ≤ 20 min for the duration of the observation period (up to 604 days) with therapeutic FIX levels for the entire observation period of up to 418 days [23]. No signs of chronic toxicity were observed. Similarly, in the case of FVIII-deficient dogs, systemic intravascular injection of HDAd expressing canine FVIII resulted in reduction of whole blood clotting time to near normal levels and low level FVIII activity for the duration of the experiment of at least 2 years [24]. However, in both these studies, acute, albeit transient, laboratory abnormalities (ALT, AST, AP, CPK, platelet counts) were observed and attributed to activation of the innate inflammatory response [23,24]. Moreover, in both dog studies above, inhibitor development were not observed. However, it should be noted that in the case of the FVIII study above, the hemophilia A dogs used are not prone to make inhibitors to cFVIII.

There has been a single case of intravascular administration of HDAd into a human patient. In this clinical trial, 4.3×10^11^ vp/kg of a HDAd expressing FVIII was intravenously injected into a hemophilia A patient [27]. This subject developed grade 3 liver toxicity, marked increase in interleukin-6 (IL-6), thrombocytopenia, and laboratory signs of disseminated intravascular coagulopathy. All these values returned to baseline by day 19 post-infusion. Unfortunately, no evidence of FVIII expression was detected. Also unfortunate is that this study has yet to be published in a peer-reviewed format so that much of the details remain unknown. Nevertheless, this acute toxicity is consistent with activation of the innate inflammatory response as described in the studies above as well as many others.

## Dose-Dependent Acute Toxicity

Although the precise mechanism(s) responsible for activation of the innate inflammatory response remains to be fully elucidated, it is mediated by the viral capsid (which is identical for all Ad-based vectors) and the severity of the response is dose-dependent. A variety of strategies have been developed to address this adverse outcome. For example, use of anti-inflammatory drugs or masking the viral capsid have shown promising results in blunting the innate inflammatory response [28–32]. Modification of the virion to permit evasion of immune cells, such as Kupffer cells of the liver, have also yielded promising results [33–34]. Because the severity of the innate response to Ad is dose-dependent, other promising strategies involve lowering the minimal effective vector dose [35–37]. One such strategy makes use of more potent expression cassettes. For example, in the case of hemophilia B, three bioengineered FIX variants with improved catalytic activity have been tested in the context of HDAd [38]. The first vector expressed R338A-FIX, a FIX variant with the arginine at position 338 changed to an alanine [39,40], which resulted in a 2.9-fold higher specific activity (IU/mg) compared with the wild-type FIX. The second vector expressed FIX_VIIEGF1_, a variant with the EGF-1 domain replaced with the EGF-1 domain from FVII [41], which resulted in a 3.4-fold increase in specific activity. The third expressed R338A + FIX_VIIEGF1_, a novel variant containing both aforementioned modifications, which resulted in a 12.6-fold increase in specific activity. High level, long-term, and stable expression of these three variants was observed in hemophilia B mice with no evidence of increased thrombogenicity compared to wild-type FIX. Thus, these bioengineered FIX variants can increase the therapeutic index of gene therapy vectors by permitting administration of lower doses to achieve the same therapeutic outcome. Various FVIII variants have also been evaluated in the context of HDAd [42]. One variant was found to be efficacious long-term at half the viral dose compared to wild-type and with reduced induction of anti-FVIII antibodies.

**Figure 3:**
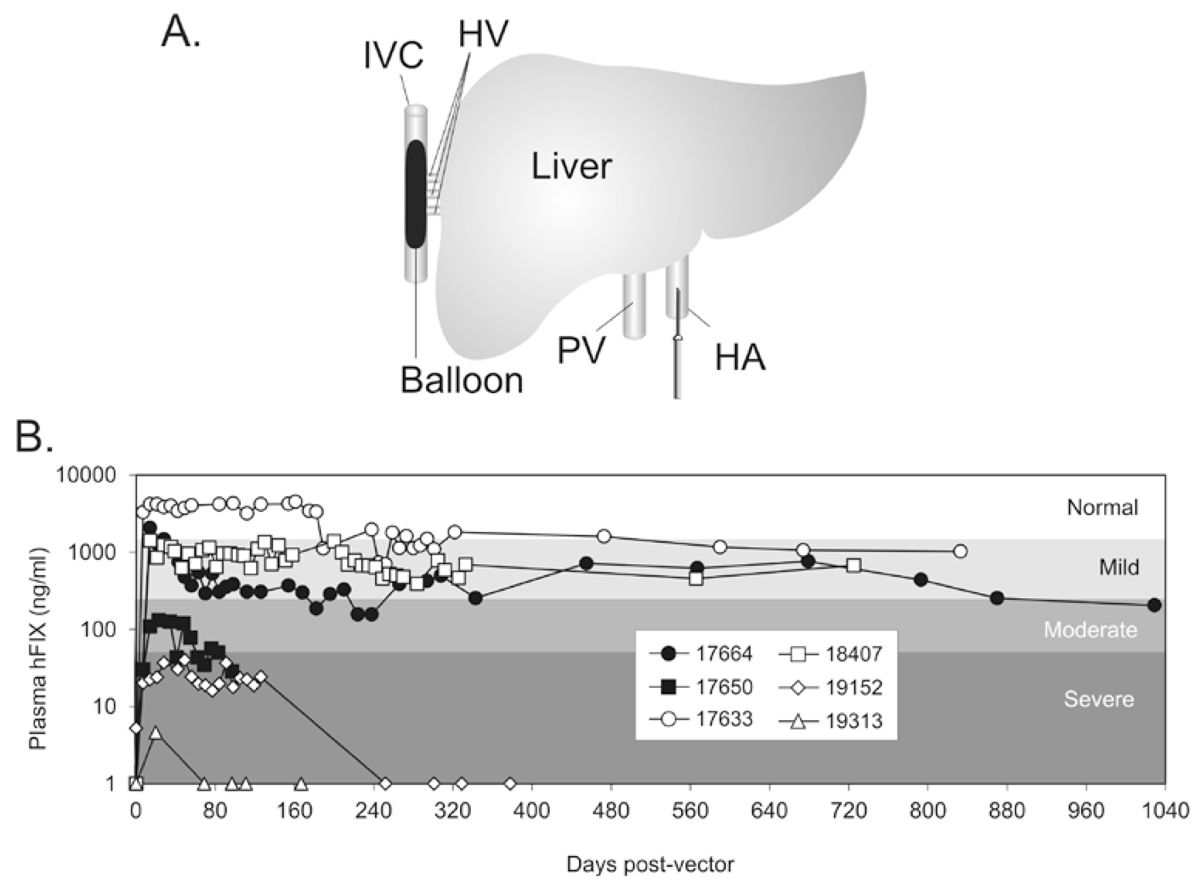
(A) A sausage-shaped balloon catheter is positioned in the inferior vena cava (IVC) under fluoroscopic guidance. Inflation of the balloon results in hepatic venous outflow occlusion from the hepatic veins (HV). The HDAd is administered by injection through a percutaneously positioned hepatic artery (HA) catheter. (B) Plasma hFIX levels in rhesus macaques. Rhesus 17664 and 17633 were injected with 1×10^12^ vp/kg; 17650 and 18407 with 1×10^11^ vp/kg; 19152 with 3×10^10^ vp/kg; 19313 with 1×10^10^ vp/kg. Normal; >30% of normal. Mild; >5% to 30 of normal. Moderate; ≥ 1% to 5% of normal. Severe; <1% of normal. Normal hFIX level is ~5,000 ng/ml. Adapted from Brunetti-Pierri et al. [46–47].

There is considerable support for the notion that extrahepatic systemic vector dissemination plays a major role in Ad-mediated acute toxicity and that preferential hepatic transduction at the expense of extrahepatic vector uptake may reduce acute toxicity [43 and references therein]. Thus, another approach to achieve dose reduction is to use routes of vector administration that permit preferential hepatocyte transduction. Several approaches have been developed for administration of HDAd that result in increased hepatocyte transduction and long-term transgene expression with reduced systemic vector dissemination thus permitting use of clinically relevant sub-toxic doses [44–46]. In one example, a balloon occlusion catheter was percutaneously positioned in the inferior vena cava to occlude hepatic venous outflow and HDAd was injected directly into the occluded liver via a percutaneously placed hepatic artery catheter (Figure 3A). This resulted in up to 80-fold improvement in hepatic transduction compared to systemic vector injection and with transgene expression sustained for up to 2.6 years [46]. This balloon catheter method was used to deliver a low dose of HDAd expression hFIX into rhesus macaques which can result in therapeutic levels of hFIX expression for up to 2.8 years post-injection (Figure 3B) [47].

Despite impressive duration of expression data in large and small animals, transgene expression from HDAd may not be permanent because the vector genome does not integrate into the host chromosome. Instead, a slow, steady decline in transgene expression has been observed and is consistent with a gradual loss of transduced hepatocytes due to physiologic turnover or loss of the episomal vector genome or a combination of both. Should transgene expression fall below therapeutic levels over time, a HDAd of a different serotype may be re-administered to overcome the neutralizing anti-Ad antibody elicited with the first administration [48,49]. An alternative approach to achieve stable transgene levels in the presence of hepatocyte turn-over is based on delivery of hyperactive Sleeping Beauty transposase system by HDAd that results in somatic integration of FIX into the hepatocyte genome [50]. By this approach, stable canine FIX expression levels from integrated vector have been observed long term both in mice and hemophilia B dogs [50].

## Outstanding Issues and Concluding Remarks

In all animal models studied to date, HDAd transduced hepatocytes (as well as all other target cell types examined) are not destroyed by an adaptive cellular immune response, thus leading to long-term transgene expression. However, whether this holds true for humans is not known, especially considering the outcomes of recent liver-directed clinical trials for FIX-deficiency with AAV vectors [51,52]. AAV vectors, like HDAd, do not contain any viral genes and mediates long-term transgene expression following hepatocyte transduction in all animal models investigated. However, in humans, AAV-mediated transgene expression from transduced hepatocytes is subject to killing by AAV-specific CTLs. The source of immunogen has been attributed to AAV capsid peptides derived directly from the injected particles [51,53,54]. Similar to AAV, the HDAd capsid proteins derived directly from the administered particles may be a source of immunogen, analogous to AAV [55,56]. In this regard, Roth et al. [57] showed that HDAd transduction of dendritic cells *in vitro* can stimulate activation of anti-Ad CD8+ T-cells. Indeed, Muruve et al. [58] showed that Ad-specific CTL were generated following intravascular administration of HDAd into mice. Similarly, Kushwah et al. [59] showed that intranasal administration of HDAd resulted in Ad-specific CD8+ T-cells. These studies show that HDAd can indeed provoke the generation of a CTL response directed against the viral proteins derived directly from the capsid independent of de novo viral protein synthesis following administration into mice. However, whether these Ad-specific CTLs will eliminate HDAd transduced cells *in vivo* remains to be shown and animal modeling do not appear useful for addressing this important issue because they were not in the case for AAV [60–62].

## References

[1] Manco-Johnson MJ, Nuss R, Geraghty S, Funk S, Kilcoyne R (1994). Results of secondary prophylaxis in children with severe hemophilia. Am J Hematol.

[2] Löfqvist T, Nilsson IM, Berntorp E, Pettersson H (1997). Haemophilia prophylaxis in young patients--a long-term follow-up. J Intern Med.

[3] Shapiro AD, Ragni MV, Valentino LA, Key NS, Josephson NC (2012). Recombinant factor IX-Fc fusion protein (rFIXFc) demonstrates safety and prolonged activity in a phase 1/2a study in hemophilia B patients. Blood.

[4] Ostergaard H, Bjelke JR, Hansen L, Petersen LC, Pedersen AA (2011). Prolonged half-life and preserved enzymatic properties of factor IX selectively PEGylated on native N-glycans in the activation peptide. Blood.

[5] Ragni MV (2004). Hemophilia gene transfer: comparison with conventional protein replacement therapy. Semin Thromb Hemost.

[6] Montgomery RR, Shi Q (2010). Alternative strategies for gene therapy of hemophilia. Hematology Am Soc Hematol Educ Program.

[7] Larsson SA (1984). Hemophilia in Sweden. Studies on demography of hemophilia and surgery in hemophilia and von Willebrand’s disease. Acta Med Scand Suppl.

[8] Sultan Y (1992). Prevalence of inhibitors in a population of 3435 hemophilia patients in France. French Hemophilia Study Group. Thromb Haemost.

[9] Shenk T, Fields BN, Knipe DM, Howley PM (1996). Adenoviridae: the viruses and their replication. Fields Viology.

[10] Graham FL, Smiley J, Russell WC, Nairn R (1977). Characteristics of a human cell line transformed by DNA from human adenovirus type 5. J Gen Virol.

[11] Kay MA, Landen CN, Rothenberg SR, Taylor LA, Leland F (1994). *In vivo* hepatic gene therapy: complete albeit transient correction of factor IX deficiency in hemophilia B dogs. Proc Natl Acad Sci U S A.

[12] Dai Y, Schwarz EM, Gu D, Zhang WW, Sarvetnick N (1995). Cellular and humoral immune responses to adenoviral vectors containing factor IX gene: tolerization of factor IX and vector antigens allows for long-term expression. Proc Natl Acad Sci U S A.

[13] Yang Y, Nunes FA, Berencsi K, Furth EE, Gonczol E (1994). Cellular immunity to viral antigens limits E1-deleted adenoviruses for gene therapy. Proc Natl Acad Sci U S A.

[14] Yang Y, Li Q, Ertl HC, Wilson JM (1995). Cellular and humoral immune responses to viral antigens create barriers to lung-directed gene therapy with recombinant adenoviruses. J Virol.

[15] Yang Y, Xiang Z, Ertl HC, Wilson JM (1995). Upregulation of class I major histocompatibility complex antigens by interferon gamma is necessary for T-cell-mediated elimination of recombinant adenovirus-infected hepatocytes *in vivo*. Proc Natl Acad Sci U S A.

[16] Kafri T, Morgan D, Krahl T, Sarvetnick N, Sherman L (1998). Cellular immune response to adenoviral vector infected cells does not require de novo viral gene expression: implications for gene therapy. Proc Natl Acad Sci U S A.

[17] Parks RJ, Chen L, Anton M, Sankar U, Rudnicki MA (1996). A helper-dependent adenovirus vector system: removal of helper virus by Cre-mediated excision of the viral packaging signal. Proc Natl Acad Sci U S A.

[18] Palmer D, Ng P (2003). Improved system for helper-dependent adenoviral vector production. Mol Ther.

[19] Reddy PS, Sakhuja K, Ganesh S, Yang L, Kayda D (2002). Sustained human factor VIII expression in hemophilia A mice following systemic delivery of a gutless adenoviral vector. Mol Ther.

[20] Balague C, Zhou J, Dai Y, Alemany R, Josephs SF (2000). Sustained high-level expression of full-length human factor VIII and restoration of clotting activity in hemophilic mice using a minimal adenovirus vector. Blood.

[21] Bristol JA, Gallo-Penn A, Andrews J, Idamakanti N, Kaleko M (2001). Adenovirus-mediated factor VIII gene expression results in attenuated anti-factor VIII-specific immunity in hemophilia A mice compared with factorVIII protein infusion. Hum Gene Ther.

[22] Hu C, Cela RG, Suzuki M, Lee B, Lipshutz GS (2011). Neonatal helper-dependent adenoviral vector gene therapy mediates correction of hemophilia A and tolerance to human factor VIII. Proc Natl Acad Sci U S A.

[23] Brunetti-Pierri N, Nichols TC, McCorquodale S, Merricks E, Palmer DJ (2005). Sustained phenotypic correction of canine hemophilia B after systemic administration of helper-dependent adenoviral vector. Hum Gene Ther.

[24] McCormack WM, Seiler MP, Bertin TK, Ubhayakar K, Palmer DJ (2006). Helper-dependent adenoviral gene therapy mediates long-term correction of the clotting defect in the canine hemophilia A model. J Thromb Haemost.

[25] Brown BD, Shi CX, Powell S, Hurlbut D, Graham FL (2004). Helper-dependent adenoviral vectors mediate therapeutic factor VIII expression for several months with minimal accompanying toxicity in a canine model of severe hemophilia A. Blood.

[26] Ehrhardt A, Xu H, Dillow AM, Bellinger DA, Nichols TC (2003). A gene-deleted adenoviral vector results in phenotypic correction of canine hemophilia B without liver toxicity or thrombocytopenia. Blood.

[27] White GI, Monahan PE (2005). Gene therapy for hemophilia A.

[28] De Geest B, Snoeys J, Van Linthout S, Lievens J, Collen D (2005). Elimination of innate immune responses and liver inflammation by PEGylation of adenoviral vectors and methylprednisolone. Hum Gene Ther.

[29] Seregin SS, Appledorn DM, McBride AJ, Schuldt NJ, Aldhamen YA (2009). Transient pretreatment with glucocorticoid ablates innate toxicity of systemically delivered adenoviral vectors without reducing efficacy. Mol Ther.

[30] Yotnda P, Chen DH, Chiu W, Piedra PA, Davis A (2002). Bilamellar cationic liposomes protect adenovectors from preexisting humoral immune responses. Mol Ther.

[31] Croyle MA, Chirmule N, Zhang Y, Wilson JM (2002). PEGylation of E1-deleted adenovirus vectorsallowssignificantgene expression on readministration to liver. Hum Gene Ther.

[32] Hofherr SE, Mok H, Gushiken FC, Lopez JA, Barry MA (2007). Polyethylene glycol modification of adenovirus reduces platelet activation, endothelial cell activation, and thrombocytopenia. Hum Gene Ther.

[33] Prill JM, Espenlaub S, Samen U, Engler T, Schmidt E (2011). Modifications of adenovirus hexon allow for either hepatocyte detargeting or targeting with potential evasion from Kupffer cells. Mol Ther.

[34] Khare R, May SM, Vetrini F, Weaver EA, Palmer D (2011). Generation of a Kupffer cell-evading adenovirus for systemic and liver-directed gene transfer. Mol Ther.

[35] Jacobs F, Feng Y, Van Craeyveld E, Lievens J, Snoeys J (2009). Species differences in hepatocyte-directed gene transfer: implications for clinical translation. Curr Gene Ther.

[36] Vetrini F, Brunetti-Pierri N, Palmer DJ, Bertin T, Grove NC (2010). Vasoactive intestinal peptide increases hepatic transduction and reduces innate immune response following administration of helper-dependent Ad. Mol Ther.

[37] Piccolo P, Vetrini F, Mithbaokar P, Grove NC, Bertin T (2013). SR-A and SREC-I Are Kupffer and Endothelial Cell Receptors for Helper-dependent Adenoviral Vectors. Mol Ther.

[38] Brunetti-Pierri N, Grove NC, Zuo Y, Edwards R, Palmer D (2009). Bioengineered factor IX molecules with increased catalytic activity improve the therapeutic index of gene therapy vectors for hemophilia B. Hum Gene Ther.

[39] Chang J, Jin J, Lollar P, Bode W, Brandstetter H (1998). Changing residue 338 in human factor IX from arginine to alanine causes an increase in catalytic activity. J Biol Chem.

[40] Schuettrumpf J, Herzog RW, Schlachterman A, Kaufhold A, Stafford DW (2005). Factor IX variants improve gene therapy efficacy for hemophilia B. Blood.

[41] Chang JY, Monroe DM, Stafford DW, Brinkhous KM, Roberts HR (1997). growth factor-like domain of factor IX with that of Replacingthefirst factor VII enhances activity *in vitro* and in canine hemophilia B. J Clin Invest.

[42] Cerullo V, Seiler MP, Mane V, Cela R, Clarke C (2007). Correction of murine hemophilia A and immunological differences of factor VIII variants delivered by helper-dependent adenoviral vectors. Mol Ther.

[43] Brunetti-Pierri N, Palmer DJ, Mane V, Finegold M, Beaudet AL (2005). Increased hepatic transduction with reduced systemic dissemination and proinflammatory cytokines following hydrodynamic injection of helper-dependent adenoviral vectors. Mol Ther.

[44] Brunetti-Pierri N, Ng T, Iannitti DA, Palmer DJ, Beaudet AL (2006). Improved hepatic transduction, reduced systemic vector dissemination, and long-term transgene expression by delivering helper-dependent adenoviral vectors into the surgically isolated liver of nonhuman primates. Hum Gene Ther.

[45] Brunetti-Pierri N, Stapleton GE, Palmer DJ, Zuo Y, Mane VP (2007). Pseudo-hydrodynamic delivery of helper-dependent adenoviral vectors into non-human primates for liver-directed gene therapy. Mol Ther.

[46] Brunetti-Pierri N, Stapleton GE, Law M, Breinholt J, Palmer DJ (2009). Efficient, long-term hepatic gene transfer using clinically relevant HDAd doses by balloon occlusion catheter delivery in nonhuman primates. Mol Ther.

[47] Brunetti-Pierri N, Liou A, Patel P, Palmer D, Grove N (2012). Balloon catheter delivery of helper-dependent adenoviral vector results in sustained, therapeutic hFIX expression in rhesus macaques. Mol Ther.

[48] Kim IH, Jozkowicz A, Piedra PA, Oka K, Chan L (2001). Lifetime correction of genetic deficiency in mice with a single injection of helper-dependent adenoviral vector. Proc Natl Acad Sci U S A.

[49] Morral N, O’Neal W, Rice K, Leland M, Kaplan J (1999). Administration of helper-dependent adenoviral vectors and sequential delivery of different vector serotype for long-term liver-directed gene transfer in baboons. Proc Natl Acad Sci U S A.

[50] Hausl MA, Zhang W, Muther N, Rauschhuber C, Franck HG (2010). Hyperactive sleeping beauty transposase enables persistent phenotypic correction in mice and a canine model for hemophilia B. Mol Ther.

[51] Manno CS, Pierce GF, Arruda VR, Glader B, Ragni M (2006). Successful transduction of liver in hemophilia by AAV-Factor IX and limitations imposed by the host immune response. Nat Med.

[52] Nathwani AC, Tuddenham EG, Rangarajan S, Rosales C, McIntosh J (2011). Adenovirus-associated virus vector-mediated gene transfer in hemophilia B. N Engl J Med.

[53] Mingozzi F, Maus MV, Hui DJ, Sabatino DE, Murphy SL (2007). CD8(+) T-cell responses to adeno-associated virus capsid in humans. Nat Med.

[54] Pien GC, Basner-Tschakarjan E, Hui DJ, Mentlik AN, Finn JD (2009). Capsid antigen presentation flags human hepatocytes for destruction after transduction by adeno-associated viral vectors. J Clin Invest.

[55] Smith CA, Woodruff LS, Kitchingman GR, Rooney CM (1996). Adenovirus-pulsed dendritic cells stimulate human virus-specific T-cell responses *in vitro*. J Virol.

[56] Molinier-Frenkel V, Gahery-Segard H, Mehtali M, Le Boulaire C, Ribault S (2000). Immune response to recombinant adenovirus in humans: capsid components from viral input are targets for vector-specific cytotoxic T lymphocytes. J Virol.

[57] Roth MD, Cheng Q, Harui A, Basak SK, Mitani K (2002). Helper-dependent express transgenes in human dendritic cells but adenoviral vectors efficiently still stimulate antiviral immune responses. J Immunol.

[58] Muruve DA, Cotter MJ, Zaiss AK, White LR, Liu Q (2004). Helper-dependent adenovirus vectors elicit intact innate but attenuated adaptive host immune responses *in vivo*. J Virol.

[59] Kushwah R, Cao H, Hu J (2008). Characterization of pulmonary T cell response to helper-dependent adenoviral vectors following intranasal delivery. J Immunol.

[60] Wang L, Figueredo J, Calcedo R, Lin J, Wilson JM (2007). Cross-presentation of adeno-associated virus serotype 2 capsids activates cytotoxic T cells but does not render hepatocytes effective cytolytic targets. Hum Gene Ther.

[61] Li H, Murphy SL, Giles-Davis W, Edmonson S, Xiang Z (2007). Pre-existing AAV capsid-specific CD8+ T cells are unable to eliminate AAV-transduced hepatocytes. Mol Ther.

[62] Li H, Lin SW, Giles-Davis W, Li Y, Zhou D (2009). A preclinical animal model to assess the effect of pre-existing immunity on AAV-mediated gene transfer. Mol Ther.

[63] Lozier JN, Metzger ME, Donahue FE, Morgan RA (1999). Adenovirus-Mediated Expression of Human Coagulation Factor IX in the Rhesus Macaque Is Associated With Dose-Limiting Toxicity. Blood.

